# Surgical Treatment for Biliary Carcinoma Arising After Pancreatoduodenectomy

**DOI:** 10.1155/1998/93740

**Published:** 1998

**Authors:** Hitoshi Seki, Shinichi Miyagawa, Akira Kobayashi, Seiji Kawasaki

**Affiliations:** First Department of Surgery Shinshu University School of Medicine Matsumoto Japan

## Abstract

The clinicopathological features and surgical treatment of biliary carcinoma around the major hepatic duct confluence arising after pancreatoduodenectomy (PD) due to initial bile duct carcinoma are described in three patients. Occurrence of biliary carcinoma more than 12 years after initial surgery and a histological finding of cholangiocellular carcinoma mixed with hepatocellular carcinoma suggested metachronous incidence of biliary carcinoma after PD. Extended right hemihepatectomy with complete removal of the residual extrahepatic bile duct and segmental, resection of the jejunal loop were carried out safely without operative death or severe postoperative complications. Two patients  died of tumor recurrence 6 months after surgery, and the remaining patient is currently living a normal life without evidence of recurrence 17 months after surgery. These surgical procedures are a therapeutic option in patients with biliary  carcinoma around the major hepatic duct confluence arising after PD.


*HPB Surgery*, 1998, Vol. 10, pp. 395-397
Reprints available directly from the publisher
Photocopying permitted by license only

© 1998 OPA (Overseas Publishers Association)
Amsterdam B.V. Published under license
under the Harwood Academic Publishers imprint,
part of The Gordon and Breach Publishing Group.
Printed in India.

*Case Report*

### Surgical Treatment for Biliary Carcinoma Arising After Pancreatoduodenectomy

HITOSHI SEKI[a], SHINICHI MIYAGAWA[a,*], AKIRA KOBAYASHI[a] and SEIJI KAWASAKI[a]

[a]*First Department of Surgery, Shinshu University, School of Medicine, Matsumoto, Japan*

*(Received 28 November 1996)*

**The clinicopathological features and surgical treatment of biliary carcinoma around the major hepatic duct confluence arising after pancreatoduodenectomy (PD) due to initial bile duct carcinoma are described in three patients. Occurrence of biliary carcinoma more than 12 years after initial surgery and a histological finding of cholangiocellular carcinoma mixed with hepatocellular carcinoma suggested metachronous incidence of biliary carcinoma after PD. Extended right hemihepatectomy with complete removal of the residual extrahepatic bile duct and segmental resection of the jejunal loop were carried out safely without operative death or severe postoperative complications. Two patients died of tumor recurrence 6 months after surgery, and the remaining patient is currently living a normal life without evidence of recurrence 17 months after surgery. These surgical procedures are a therapeutic option in patients with biliary carcinoma around the major hepatic duct confluence arising after PD.**

*Keywords:* Biliary carcinoma arising after pancreatoduodenectomy, surgical treatment, metachronous incidence

### INTRODUCTION

Surgical treatment for biliary carcinoma arising around the major hepatic duct confluence in patients who had undergone pancreatoduodenectomy (PD) due to initial bile duct carcinoma has been reported only rarely [1]. This article describes three patients with biliary carcinoma arising after PD, and discusses their clinicopathological characteristics and surgical treatment.

### PATIENT PROFILE

In the three patients in whom PD had been performed due to initial bile duct carcinoma, complete removal of the main tumor including

*Correspondence: SHINICHI MIYAGAWA, First Department of Surgery, Shinshu University, School of Medicine, 3-1-1 Asahi, Matsumoto 390, Japan. Tel. 81 263 37 2654, Fax. 81 263 35 1282.

the regional lymph nodes had been achieved. They had suffered several episodes of acute cholangitis and/or obstructive jaundice before admission. Direct cholangiography revealed strictures in the residual biliary tree. In these three patients, the time from initial surgery to presentation with further tumor was 50, 70 and 153 months, respectively. Preoperative imaging examinations showed biliary carcinoma extending from the hepaticojejunal anastomosis to both the right and left hepatic ducts. Therefore residual extrahepatic bile duct resection with concomitant hepatectomy was chosen. A metastatic liver tumor located in segment 3 and residual gastric cancer were also detected in one of the patients (No. 2) (Tab. I).

### RESULTS

Operation time and operative blood loss are shown in Table I. Postoperatively, an abdominal abscess developed in one patient (No. 2), but this was treated successfully without re-laparotomy. Histological examination of the resected specimens showed cholangiocarcinoma in two patients and cholangiocellular carcinoma mixed with hepatocellular carcinoma in the other (No. 1). One patient (No. 2) had a surgical margin microscopically positive for carcinoma at the left hepatic duct due to subepithelial spread of the carcinoma. Metastasis to the lymph nodes in the jejunal loop, which had been arised for hepaticojejunostomy, was detected in one patient (No. 3). There was no operative or hospital mortality. Two patients (Nos. 1 and 2) died of tumor recurrence 6 months after surgery, and the remaining patient (No. 3) is currently living a normal life without evidence of recurrence 17 months after surgery.

### DISCUSSION

Few reports have described metachronous carcinoma in the biliary tree. Among the present three patients, occurrence of metachronous biliary carcinoma was strongly suspected in two, because one (No. 1) had cholangiocellular carcinoma mixed with hepatocellular carcinoma, which was histologically different from the initial tumor, and the other (No. 2) had biliary carcinoma arising more than 12 years after removal of the initial malignancy. In the remaining patient (No. 3), the possibility of very slow recurrence of the primary lesion could not be ruled out in view of the histological features of the first and second resected specimens, even though more than five years had passed since the initial operation.

Surgical resection is the only curative treatment for biliary carcinoma [2]. The role of repeated resection for treatment of biliary carcinoma arising after PD is not well documented [1, 3]. In most patients with biliary carcinoma arising after PD, previous surgery

TABLE I Characteristics of the three patients with metachronous biliary carcinoma

| No. | Age | Sex | Histology of initial ca. | Second ca. location | Time (M) | Op. | Op.T (min) | Op. B (ml) | Histology of second ca. | Outcome |
|---|---|---|---|---|---|---|---|---|---|---|
| 1 | 75 | M | Tub. | RHD to IHBD | 50 | ERH | 650 | 910 | CCC+HCC | D (6 M) |
| 2 | 68 | M | Pap-tub | Biliary con. | 153 | ERH+$\alpha$ | 1065 | 2460 | Pap-tub. | D (6 M) |
| 3 | 69 | F | Pap-tub. | Biliary con. | 70 | ERH | 700 | 738 | Pap. | A (17 M) |

No., number of patient; Ca. carcinoma; Time (M), time from initial surgery to presentation with further tumor (months); Op, operative procedure; Op.T. (min), operation time (minutes); Op. B. (ml), operative blood loss (ml); RHD, right hepatic duct; IHBD, intrahepatic bile duct; ERH, extended right hemihepatectomy; Biliary con., major biliary confluence; Tub., tubular adenocarcinoma; Pap-tub., papillo-tubular adenocarcinoma; Pap., papillary adenocarcinoma; $\alpha$, partial gastrectomy and limited resection of segment 3 due to a metastatic liver tumor; CCC, cholangiocellular carcinoma; HCC, hepatocellular carcinoma; D (6 M), died at 6 months; A (17 M), disease-free at 17 months.

and the spread of the tumor preclude re-resection, and instead percutaneous external drainage is chosen. Analysis of several large series published in recent years does not provide information on the feasibility of repeated resection [1]. Radical extended resection, such as extended right hemihepatectomy, for carcinoma of the biliary confluence gives better survival figures than local resection [4, 5].

In the present three patients, concomitant extended right hemihepatectomy was chosen because the extrahepatic biliary carcinoma was located around the major hepatic duct confluence in two patients (Nos. 2 and 3), and the cancer arose from the intrahepatic bile duct and extended to the major hepatic duct confluence in the other patient (No. 1). The operations were carried out safely, and one patient (No. 3) is now enjoying a normal life without recurrence more than 17 months after surgery. Complete removal of the residual extrahepatic bile duct and segmental resection of the jejunal loop with concomitant major hepatectomy is a therapeutic option in patients with biliary carcinoma around the major hepatic duct confluence arising after PD.

### References

[1] Tarragona, M., Zografos, G. and Habib, N. (1993). Liver resection for recurrent hilar cholangiocarcinoma. *Br. J. Surg.*, **80**, 1433.

[2] Reding, R., Buard, J. L., Lebeau, G. and Launois, B. (1991). Surgical management of 552 carcinomas of extrahepatic bile duct (gallbladder and periampullary tumors excluded). *Ann. Surg.*, **213**, 236-241.

[3] Gertsch, P., Thomas, P., Bear, H., Lerut, J., Zimmermann, A. and Blumgart, L. H. (1990). Multiple tumors of the biliary tract. *Am. J. Surg.*, **159**, 386-388.

[4] Bismuth, H., Nakache, R. and Diamond, T. (1992). Managment strategies in resection for hilar cholangiocarcinoma. *Ann. Surg.*, **215**, 31-37.

[5] Miyagawa, S., Makuuchi, M. and Kawasaki, S. (1995). Outcome of extended right hepatectomy after biliary drainage in hilar bile duct cancer. *Arch. Surg.*, **130**, 759-763.

### COMMENTARY

The above manuscript describes an aggressive approach (extended right hepatectomy) for recurrent cholangiocarcinoma following pancreaticoduodenopathy. It is always attractive for surgeons to consider long-term survivors following primary surgery for repeated surgical resections. Those patients considered for repeat resection had survival ranging from 50 to 153 months following the initial resection. These patients have probably selected themselves due to the relative benign course of their post-operative survival.

Repeat resection can be very difficult and may necessitate vascular reconstruction. In this series the operative time ranged from 11 to 17 hours. The authors are to be congratulated on their results as there was no operative or hospital mortality and minimal morbidity.

Unfortunately, two patients died after 6 months. However, the third patient profitted from repeated resection with 17 months survival without recurrence. It is not clear at this stage, how the patients most suitable for resection were selected. It is also not clear from this study why the third patient survived and the other died within a short period of the recurrence.

Studies of tumour biology such as the presence of gene mutation and genomic instability may enable the HPB surgeon to select those patients with less aggressive phenotype that might benefit from aggressive surgery.

Mr. N. Habib
Royal Postgraduate Medical School
Hammersmith Hospital
Du Cane Road
London W12 0NN

